# Preliminary validation of the Bulgarian SCCAN: initial reliability, validity, and screening accuracy across neurological populations

**DOI:** 10.3389/fpsyg.2026.1846359

**Published:** 2026-07-06

**Authors:** Kostadin Chompalov, Gregory J. Spray, Dobrinka Georgieva

**Affiliations:** 1 Faculty of Medicine, Medical University of Plovdiv, Plovdiv, Bulgaria; 2 Department of Speech, Language, and Hearing Sciences, Auburn University, Auburn, Alabama; 3 Research Institute at Medical University of Plovdiv, Plovdiv, Bulgaria

**Keywords:** Alzheimer’s disease, cognitive-communicative disorders, ischemic stroke, mild cognitive impairment, neuropsychological assessment, SCCAN-B

## Abstract

**Purpose:**

Bulgaria currently lacks standardized instruments for assessing cognitive-communicative abilities in adults with neurological disorders. This study reports preliminary validation evidence for the Bulgarian version of the Scales of Cognitive and Communicative Ability for Neurorehabilitation (SCCAN-B).

**Methods:**

Eighty-nine Bulgarian-speaking adults were assessed at a tertiary university hospital in Plovdiv, Bulgaria. The main analytic sample included four separate groups: neurotypical controls, individuals with mild cognitive impairment (MCI), Alzheimer’s disease (AD) dementia, and ischemic stroke. All participants had previously received a formal diagnosis prior to being classified within one of these four groups. After informed consent was obtained, participants completed the SCCAN-B and the validated Bulgarian version of the Mini-Mental State Examination (MMSE-B). Analyses examined internal consistency, convergent validity with MMSE-B, known-groups differences, and preliminary screening classification using receiver operating characteristic analysis.

**Results:**

The SCCAN-B showed high internal consistency across the eight performance scales, α = 0.931. The SCCAN-B total score correlated strongly with MMSE-B scores, ρ = 0.84, *p* < 0.001. Significant group differences were observed for the SCCAN-B total score and all performance scales, with the largest impairments in the Alzheimer’s disease group. ROC analysis distinguishing controls from the main clinical groups yielded an AUC of 0.850. A preliminary SCCAN-B total cut-off of 80 showed 70.7% sensitivity and 87.1% specificity.

**Conclusion:**

Findings provide preliminary evidence that the SCCAN-B is a clinically promising screening instrument for identifying cognitive-communicative difficulties in Bulgarian-speaking neurological populations. However, the results should be interpreted cautiously because of the modest sample size, clinical heterogeneity, limited control of confounding variables, and the need for further normative and cross-cultural validation work.

## Introduction

### Background and rationale

Cognitive-communicative disorders are frequently caused by neurological disorders such as Alzheimer’s disease (AD), cerebrovascular accident (i.e., stroke), and mild cognitive impairment (MCI). Individuals with cognitive-communication disorders typically exhibit deficits in attention, orientation, memory, language expression and comprehension, reading, writing, and problem-solving. These impairments can disrupt rehabilitation outcomes, leading to loss of autonomy, social integration, and overall quality of life (Chiaravalloti and DeLuca, 2008; Bayles et al., 2018). Comprehensive evaluation of cognitive-communicative disorders is therefore critical for differential diagnosis and therapy planning (Hillis and Heidler, 2002; Hobden et al., 2023).

International evidence supports the use of valid and reliable methods to properly assess clinical populations. Recent studies have highlighted several key characteristics of high-quality cognitive-communication assessments. First, despite the multiplicity of available tools, relatively few instruments demonstrate strong psychometric standards (Verhoeks et al., 2024). Second, assessments should adopt a patient-centered approach that reflects authentic real-world activities and functional demands (Togher et al., 2023). Qualitative research further indicates that patients value empathy, clarity, and constructive feedback during cognitive assessment, emphasizing that assessment tools and administration styles should be sensitive to patients’ experiences and emotional wellbeing (Hobden et al., 2023).

Despite these well-established criteria, access to comprehensive cognitive-communication assessment tools remains limited in many linguistic and cultural contexts, including Bulgaria. Aside from screening instruments such as the Bulgarian version of the Mini-Mental State Examination (MMSE-B; Folstein et al., 1975; Raycheva et al., 2013), there are currently no standardized Bulgarian-language measures that comprehensively assess cognitive-communication abilities in adults. Existing tools for other clinical groups (e.g., children) focus primarily on speech and language development rather than cognitive-communication function. This gap has prevented clinicians from fully assessing cognitive-communication abilities in Bulgarian patients, particularly when multiple domains are affected.

The Scales of Cognitive and Communicative Ability for Neurorehabilitation (SCCAN; Milman and Holland, 2012) is a standardized, computerized battery that is administered in English to assess cognition and communication across all neurological groups (Milman et al., 2008). The SCCAN demonstrates strong psychometric properties, including good internal consistency and construct validity, allowing for detailed profiling of both preserved and impaired abilities. As such, the SCCAN supports differential diagnosis and the development of individualized, evidence-based treatment plans for individuals with a wide range of cognitive-communication deficits.

### Need for adaptation and validation

Questionnaires such as the MMSE-B are convenient for broad cognitive screening; however, they do not provide detailed domain-specific cognitive-communication profiles (Folstein et al., 1975). While the SCCAN has the potential to address this limitation, its use in Bulgarian clinical practice requires systematic translation, cultural adaptation, and validation. Cross-cultural adaptation of assessment instruments involves more than direct translation; it requires conceptual and semantic equivalence, avoidance of cultural bias, and preservation of score meaning. Established guidelines, including those proposed by Beaton et al. (2000) and the International Test Commission [ITC] (2018), emphasize procedural rigor, identification of culture-bound items, and verification of functional equivalence.

Furthermore, when assessments are applied in new linguistic or cultural contexts, empirical validation is required to establish reliability, validity, and screening utility. The World Health Organization [WHO] (2021) reports a global increase in neurological disorders associated with cognitive-communication impairments, underscoring the need for standardized, comprehensive assessment tools in multiple languages. Taken together, these considerations highlight the necessity of adapting and validating the Bulgarian version of the SCCAN prior to its clinical implementation.

### Aims of the study

The overall aim of this study was to provide preliminary, initial-validation evidence for the Bulgarian version of the Scales of Cognitive and Communicative Ability for Neurorehabilitation (SCCAN-B). Specifically, we evaluated the SCCAN-B with respect to: (1) internal consistency of the eight performance scales; (2) convergent validity through association with the MMSE-B; (3) known-groups validity through comparison of neurotypical controls and clinical neurological groups; and (4) preliminary screening utility using ROC analysis and cut-off classification.

## Materials and methods

### Participants

A total of 98 Bulgarian-speaking adults were included in this study. Data were collected between 2023 and 2025 in the Department of Neurology, St. George University Hospital, Plovdiv, Bulgaria. Participants were identified through hospital records and routine neurological evaluations. The sample should be considered a convenience clinical sample from a tertiary university hospital setting rather than a population-representative Bulgarian sample. The sample size was considered appropriate for an initial validation and clinical feasibility study aimed at estimating preliminary reliability, convergent validity, and known-groups differences. However, it was not intended to provide full normative standardization or stable disorder-specific cut-off values. Clinical participants were recruited consecutively during routine inpatient or outpatient neurological assessment when they met the diagnostic criteria for one of the target groups: Alzheimer’s disease (AD) dementia, mild cognitive impairment (MCI), acute ischemic stroke, or multiple sclerosis (MS). Potential participants were identified by the clinical neurology team based on medical history, neurological examination, available neuroimaging, and established diagnostic criteria. Healthy controls were recruited from community-dwelling volunteers and accompanying individuals without known neurological disease, cognitive complaints, or functional dependence. All participants were native Bulgarian speakers and were included only if they had sufficient sensory, motor, and communicative abilities to complete the assessment. Written informed consent was received from all participants prior to testing and all research procedures complied with the Declaration of Helsinki. The Ethics Committee of the Medical University of Plovdiv formally approved the analysis and the publishing of the data collected (Protocol No. 4/10.04.2025; Decision R-KNE-20/16.07.2025).

Due to the requirements of the study, all participants were native-Bulgarian speakers and indicated Bulgarian as their dominant language. In addition, participants were required to have normal (or corrected to normal) vision, hearing, and sufficient motor function to participate in testing. Exclusion criteria included severe aphasia, apraxia, acute psychiatric disorders, or serious medical conditions that could prevent valid assessment (i.e., participant group classification). Language abilities were evaluated clinically during neurological examination and informal conversation to ensure adequate comprehension and expression. No formal aphasia screening test was administered.

After informed consent was obtained, participants were assigned to one of five predefined groups according to clinical diagnosis: AD (*n* = 21), MCI (*n* = 13), acute ischemic stroke (*n* = 24), controls (*n* = 31), and MS (*n* = 9). The MS subgroup (*n* = 9) was retained for exploratory descriptive reporting only because of its small sample size and potential treatment-related confounding during relapse management. Therefore, the main analytic sample included 89 participants from the control, MCI, AD, and acute ischemic stroke groups. Participants in the AD group were diagnosed with AD based on the DSM-5 criteria (American Psychiatric Association [APA], 2013) and recommendations outlined by the National Institute on Aging and the Alzheimer’s Association (NIA-AA) guidelines (McKhann et al., 2011). As such, the diagnosis included verification by history in patients and informants, objective proof for cognitive decline, functional impairment in activities of daily living, and neuroimaging (MRI or CT) to exclude other explanations.

Participants in the MCI group had subjective and objective cognitive decline, intact quotidian functionality, and absence of dementia. These criteria were defined by Petersen et al. (2014).

The participants in the ischemic stroke group were evaluated in the initial post-acute period. Scores on the National Institute of Health Stroke Scale (NIHSS; Brott et al., 1989) ranged from 1 to 8, and Glasgow–Liège Coma Scale (Born et al., 1985; Born, 1988) scores were greater than 19. Individuals with past history of stroke, psychiatric conditions, or other confounding states, were excluded.

To be included in the control group, participants were required to have no history of neuropsychiatric disease, no complaints of memory or cognition, typical communication skills, and be living independently.

Table 1 shows demographic characteristics (i.e., age, sex, and years of education) for each group.

**TABLE 1:** Demographic characteristics by group.

Group	*N*	Mean age (years) ± SD (range)	Sex (male/female)	Education (years) ± SD
Control group	31	66.8 ± 5.6 (57–78)	8/23	13.4 ± 2.2
Mild cognitive Impairment	13	70.8 ± 7.6 (54–78)	7/6	14.7 ± 1.9
Alzheimer’s disease	21	72.6 ± 7.6 (58–84)	11/10	12.2 ± 2.4
Ischemic stroke	24	67.9 ± 12.7 (36–93)	14/10	12.2 ± 2.5
*p*-value, main analytic groups		0.047	0.048	0.005

The table provides demographic characteristics for each group of participants. *p*-values refer to comparisons among the four main analytic groups. Age and education were compared using the Kruskal–Wallis test. Sex distribution was compared using the chi-square test.

### Instruments

Cognitive-communicative performance was assessed using the SCCAN-B and the MMSE-B (Folstein et al., 1975; Raycheva et al., 2013). The SCCAN is a standardized adult battery for the evaluation of neurological disorders (Milman et al., 2008) consisting of eight cognitive-communication performance scales (i.e., Oral Expression, Orientation, Memory, Auditory Comprehension, Reading Comprehension, Writing, Attention, and Problem Solving), calculated from 12 tasks/subtests. The SCCAN-B Total Raw Score (hereafter, SCCAN-B total score) ranges from 0 to 94, where a higher score indicates higher performance. The degree of severity is graded using the following scale: severe impairment (0–46), moderate impairment (47–68), mild impairment (69–86), or typical functioning (87–94).

#### Translation and cultural adaptation of the SCCAN-B

The SCCAN-B was developed through a structured translation and cultural adaptation process informed by international recommendations for cross-cultural test adaptation, including the principles of semantic, conceptual, cultural, and functional equivalence (Beaton et al., 2000; International Test Commission [ITC], 2018). The adaptation process included several steps. First, the SCCAN materials were translated from English into Bulgarian by two independent translators with experience in medical terminology and medical speech-language pathology. Second, the two Bulgarian translations were compared by the research team and clinical experts. Any disagreements were discussed until one agreed Bulgarian version was produced. Third, this version was back-translated into English to check semantic correspondence with the original version. Finally, the adapted Bulgarian version was pilot-tested to evaluate comprehension, cultural applicability, and technical feasibility.

The adaptation was guided by the biopsychosocial framework of the International Classification of Functioning, Disability and Health (World Health Organization [WHO], 2001), which emphasizes not only impairment but also activity, participation, and contextual factors relevant to real-life communication. Culture-sensitive content from the original SCCAN was reviewed to identify items requiring modification for Bulgarian-speaking adults. Several targeted adaptations were introduced to preserve functional equivalence in the Bulgarian context. These included adapting the phonemic fluency task from the English letter “f” to the Bulgarian letter “M,” replacing the U.S. map with a map of Bulgaria, replacing the U.S. emergency number “911” with “112,” adapting U.S.-specific medication names to names familiar in Bulgarian clinical practice, replacing the idiom “She has a green thumb” with the Bulgarian idiom “Тя има златни ръце,” (English translation: “she has golden hands”) and adapting date and currency formats to Bulgarian conventions.

The language and cultural modifications did not change the overall structure or intended cognitive-communicative demands of the tasks, but were introduced to improve cultural accessibility and preserve functional equivalence. A structured summary of the Bulgarian translation and cultural adaptation procedure is provided in Supplementary material 1. Because the SCCAN is a copyrighted instrument owned by PRO-ED, Inc., Supplementary material does not reproduce test items, scoring materials, stimulus content, or examiner instructions.

The Bulgarian version of the Mini-Mental State Examination (Folstein et al., 1975; Raycheva et al., 2013) was used as a global cognitive screening measure. The MMSE-B has been validated for use in Bulgarian older adults and remains widely used in Bulgarian neurological practice. The second edition of the Mini-Mental State Examination (Folstein et al., 2010) was not used in the present study because a validated Bulgarian version was not available to the authors for this research. Importantly, the MMSE-B was used as an external global cognitive screening reference and not as a comprehensive neuropsychological or cognitive-communicative assessment. Therefore, correlations between SCCAN-B and MMSE-B were interpreted as preliminary evidence of convergent validity with a global cognitive screening measure.

### Procedure

All testing was done individually in a quiet, well-lit room. The order of test administration was standardized between participants: the SCCAN-B was administered first, followed by the MMSE-B. The order was consistent between participants for pragmatic clinical reasons and to insure procedural consistency across participants. Thus, randomization of test order was not performed. Sessions ranged from 35 to 60 min, and brief rest breaks were offered if needed. All participants were administered the full SCCAN-B, with standard discontinuation (tailored-testing) procedures applied within subtests in accordance with the original SCCAN guidelines.

A practicing, masters-level student in medical speech-language pathology at Medical University of Plovdiv gave the SCCAN-B to five participants in the stroke group under guidance and supervision of the first author (KC, a neurologist and practicing Bulgarian medical speech-language pathologist). All remaining test administrations were conducted by the first author (KC).

### Statistical analysis

All analyses were performed with IBM SPSS Statistics, version 22. The level of significance was set at *p* < 0.05 (two-tailed). Descriptive statistics were calculated for each group, including means, standard deviations, standard errors of the mean, medians, score ranges, and 95% confidence intervals when appropriate. Normality of distributions was tested with the Shapiro–Wilk test. Given that many variables were not normally distributed, nonparametric methods were used for most analyses.

Internal consistency of the SCCAN-B was evaluated using Cronbach’s alpha, calculated based on the eight SCCAN-B performance scale raw scores. Additional analyses examined the contribution of each performance scale to overall reliability by computing alpha values if a scale was deleted, as well as correlations between individual performance scales and the SCCAN-B total score. Convergent validity was examined using Spearman’s rank-order correlations between SCCAN-B performance scale scores, the SCCAN-B total score, and MMSE-B scores.

Finally, known-groups validity and preliminary screening utility were examined using between-group comparisons and ROC analysis, and cut-off classification. Group differences across AD, MCI, ischemic stroke, and control groups were assessed using the Kruskal–Wallis H test, followed by Dunn’s *post-hoc* pairwise comparisons with Bonferroni correction when appropriate. Effect sizes were reported in addition to *p*-values. For Kruskal–Wallis analyses, epsilon-squared values were calculated to estimate the magnitude of between-group differences. For pairwise nonparametric comparisons, effect size *r* was calculated when the relevant test statistics were available. Screening utility was evaluated using Receiver Operating Characteristic (ROC) analysis, including calculation of the area under the curve (AUC), sensitivity, specificity, and optimal cut-off values based on the Youden index. In addition to ROC analysis, cut-off classification was examined descriptively by calculating the number and percentage of participants in each group scoring below and at or above the preliminary SCCAN-B total score threshold of 80.

## Results

### Descriptive statistics

A total of 89 participants were included in the study and were separated into one of four main groups: AD (*n* = 21), MCI (*n* = 13), acute ischemic stroke (*n* = 24), and controls (*n* = 31). See Table 1.

The Kruskal–Wallis H test revealed a statistically significant difference in age across the four main analytic groups, χ^2^(3) = 7.94, *p* = 0.047. Dunn’s *post-hoc* test with Bonferroni correction showed that participants in the control group were younger than those in the AD group. A statistically significant difference was also found for years of education, χ^2^(3) = 13.04, *p* = 0.005. *Post-hoc* pairwise comparisons using Dunn’s procedure with Bonferroni correction revealed that participants with MCI (*Mdn* = 16) had significantly more years of education than participants with ischemic stroke (*Mdn* = 12) and AD (*Mdn* = 12), both *p* < 0.0083. No other pairwise differences were statistically significant. A chi-square test of independence showed a statistically significant difference in sex distribution across groups, χ^2^(3) = 7.88, *p* = 0.048, mainly reflecting a higher proportion of females in the control group compared with the clinical groups. Table 2 presents descriptive statistics for the eight SCCAN-B performance scales, the SCCAN-B total score, and the MMSE-B by group. For the SCCAN-B total score and MMSE-B, standard errors of the mean and 95% confidence intervals are also reported to provide an estimate of precision around group means.

**TABLE 2:** Mean ( ± SD) SCCAN-B total Score, SCCAN-B performance scale score, and MMSE-B score by group.

Measure	Maximum score	Control group (*n* = 31)	MCI (*n* = 13)	AD (*n* = 21)	Ischemic stroke (*n* = 24)
MMSE-B	30	29.16 ± 1.10	26.62 ± 2.60	18.81 ± 3.47	25.79 ± 5.55
SCCAN-B total score	94	85.58 ± 8.55	76.15 ± 9.24	50.38 ± 11.90	74.00 ± 20.16
Attention	16	13.45 ± 2.75	12.31 ± 2.46	8.05 ± 2.75	11.92 ± 3.90
Orientation	12	12.00 ± 0.00	11.62 ± 0.96	8.52 ± 2.58	11.12 ± 2.09
Memory	16	15.87 ± 3.20	9.85 ± 3.60	3.86 ± 1.15	10.79 ± 4.81
Writing	7	6.97 ± 0.18	6.85 ± 0.38	6.52 ± 0.98	6.21 ± 1.84
Problem solving	24	20.13 ± 3.90	18.46 ± 3.20	11.71 ± 4.56	17.92 ± 5.87
Auditory comprehension	13	11.97 ± 1.45	11.00 ± 1.78	7.33 ± 1.93	10.67 ± 2.63
Oral expression	22	18.16 ± 2.35	15.08 ± 4.25	10.24 ± 3.86	15.96 ± 4.60
Reading comprehension	12	11.10 ± 1.30	11.23 ± 1.09	8.52 ± 2.06	10.21 ± 2.86

Average scores, maximum scores, and standard deviation are presented for each group of participants. Scores are respective to the Bulgarian Mini Mental Status Exam (MMSE-B), Bulgarian Scales of Cognitive-Communicative Ability for Neurorehabilitation (SCCAN-B), and each performance scale of the SCCAN-B.

Table 3 provides standard errors of the mean and 95% confidence intervals for the SCCAN-B total score and MMSE-B by group, offering an estimate of precision around the group means.

**TABLE 3:** SCCAN-B total and MMSE-B scores by group with standard error of the mean and 95% confidence intervals.

Measure	Group	*n*	Mean	SD	SEM	95% CI
SCCAN-B total	Control	31	85.58	8.55	1.54	82.44–88.72
SCCAN-B total	MCI	13	76.15	9.24	2.56	70.57–81.73
SCCAN-B total	AD	21	50.38	11.90	2.60	44.96–55.80
SCCAN-B total	Ischemic stroke	24	74.00	20.16	4.12	65.49–82.51
MMSE-B	Control	31	29.16	1.10	0.20	28.76–29.56
MMSE-B	MCI	13	26.62	2.60	0.72	25.05–28.19
MMSE-B	AD	21	18.81	3.47	0.76	17.23–20.39
MMSE-B	Ischemic stroke	24	25.79	5.55	1.13	23.45–28.13

MCI, Mild cognitive impairment; AD, Alzheimer’s disease; SCCAN-B, Bulgarian version of the Scales of Cognitive and Communicative Ability for Neurorehabilitation; and MMSE-B, Mini-Mental Status Examination.

### Internal consistency

The SCCAN-B demonstrated a high level of internal consistency (Cronbach’s α = 0.931). The coefficient was calculated using the eight SCCAN-B subtest raw scores as input variables (i.e., one score per performance scale), based on their variance–covariance structure.

Spearman’s rank-order correlations revealed statistically significant positive relationships between each SCCAN-B performance scale and the SCCAN-B total score (all *p* < 0.001). Very strong correlations were found for Memory [*r*_*s*_(87) = 0.93, *p* < 0.001], Attention [*r*_*s*_(87) = 0.90, *p* < 0.001], Oral Expression [*r*_*s*_(87) = .86, *p* < 0.001], and Problem Solving [*r*_*s*_(87) = 0.86, *p* < 0.001]. Strong correlations were observed for Orientation [*r*_*s*_(87) = 0.76, *p* < 0.001], Auditory Comprehension [*r*_*s*_(87) = 0.76, *p* < 0.001], and Reading Comprehension [*r*_*s*_(87) = 0.75, *p* < 0.001]. A moderate correlation was found for the Writing scale (*r*_*s*_(87) = 0.53, *p* < 0.001).

Table 4 shows alpha values if a performance scale was deleted, as well as how the performance scale correlates with the SCCAN-B total score.

**TABLE 4:** Internal consistency and correlations with SCCAN-B total score.

Subtest	Mean ± SD	Alpha if subtest deleted	Spearman rho with total	*p*-value
Oral expression	15.25 ± 4.73	0.899	0.86	<0.001
Orientation	10.89 ± 2.16	0.915	0.76	<0.001
Memory	10.79 ± 5.68	0.919	0.93	<0.001
Auditory Comprehension	10.38 ± 2.64	0.908	0.76	<0.001
Reading comprehension	10.27 ± 2.22	0.916	0.75	<0.001
Writing	6.64 ± 1.11	0.932	0.53	<0.001
Attention	11.60 ± 3.66	0.899	0.90	<0.001
Problem Solving	17.30 ± 5.56	0.901	0.86	<0.001
Overall scale (Cronbach’s alpha)		0.931		

Internal consistency is shown, along with correlations between each performance scale and the SCCAN-B total score.

### Convergent validity

Convergent validity was examined by correlating SCCAN-B scores (total score and each performance scale) with MMSE-B scores. The SCCAN-B total score showed a strong positive correlation with the MMSE-B [*r*_*s*_ (87) = 0.840, *p* < 0.001]. For the SCCAN-B performance scales, the strongest correlations were observed for Memory [*r*_*s*_ (87) = 0.827, *p* < 0.001], Oral Expression [*r*_*s*_ (87) = 0.747, *p* < 0.001], Attention [*r*_*s*_ (87) = 0.74, *p* < 0.001], Orientation [*r*_*s*_ (87) = 0.711, *p* < 0.001], and Problem Solving [*r*_*s*_ (87) = 0.71, *p* < 0.001]. Significant correlations were also found for Auditory Comprehension [*r*_*s*_ (87) = 0.66, *p* < 0.001], Reading Comprehension [*r*_*s*_(87) = 0.63, *p* < 0.001], and Writing [*r*_*s*_(87) = 0.46, *p* < 0.001].

**FIGURE 1:**
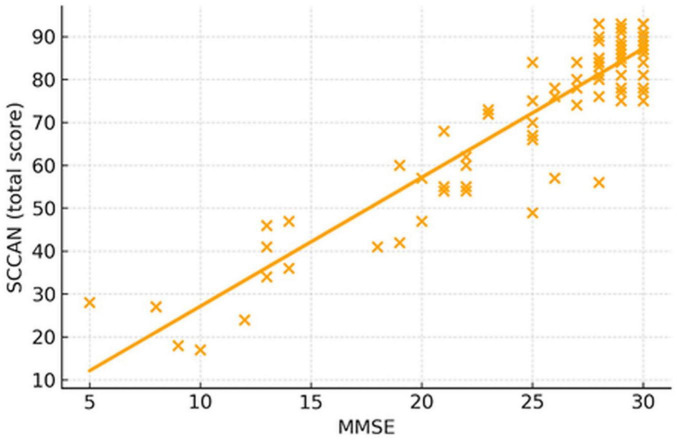
Relationship between MMSE-B and SCCAN-B (total score).

Table 5 summarizes these values. Figure 1 illustrates the association between the SCCAN-B total score and the MMSE-B.

**TABLE 5:** Convergent validity: correlations between MMSE-B scores and SCCAN-B performance scores.

Subtest	Spearman rho	*p*-value
Oral expression	0.74	<0.001
Orientation	0.73	<0.001
Memory	0.83	<0.001
Auditory comprehension	0.66	<0.001
Reading comprehension	0.63	<0.001
Writing	0.46	<0.001
Attention	0.74	<0.001
Problem solving	0.71	<0.001
SCCAN-B total	0.84	<0.001

Correlations between the MMSE-B and each SCCAN-B performance scale, along with the SCCAN-B total score.

### Group comparisons

Group differences were examined with the Kruskal–Wallis H test. Statistically significant between-group differences were found for the SCCAN-B total score [χ^2^(3) = 48.86, *p* = 1.4 × 10^–10^], and each of the SCCAN-B performance scales: Oral Expression [χ^2^(3) = 41.55, *p* = 5 × 10^–9^], Orientation [χ^2^(3) = 40.80, *p* = 7.2 × 10^–9^], Memory [χ^2^(3) = 55.91, *p* = 4.4 × 10 ^–12^], Auditory Comprehension [χ^2^(3) = 38.15, *p* = 2.6 × 10^–8^], Reading Comprehension [χ^2^(3) = 29.36, *p* = 1.89 × 10^–6^], Attention [χ^2^(3) = 35.31, *p* = 1.1 × 10^–7^], Problem Solving [χ^2^(3) = 35.23, *p* = 1.1 × 10^–7^], Writing performance scale [χ^2^(3) = 30.33, *p* = 1.2 × 10^–6^]. Effect size estimates were large for the SCCAN-B total score (ε^2^ = 0.54) and Memory (ε^2^ = 0.62), and moderate-to-large across the remaining SCCAN-B performance scales (ε^2^ = 0.31–0.45), indicating clinically meaningful group-level differences beyond statistical significance. Pairwise comparisons were performed using Dunn’s procedure with Bonferroni correction for the SCCAN-B total score and each performance scale. *Post-hoc* analysis revealed statistically significant differences in SCCAN-B total score between the control group (*Mdn* = 87) and the ischemic stroke group (*Mdn* = 79) (*p* < 0.001), between the control group (*Mdn* = 87) and the AD group (*Mdn* = 47) (*p* < 0.001), and between the mild cognitive impairment (MCI) group (*Mdn* = 76) and the AD group (*Mdn* = 47) (*p* < 0.001).

#### Oral expression

*Post-hoc* analysis revealed statistically significant differences in oral expression performance scores between the control group (*Mdn* = 19) and the AD group (*Mdn* = 9) (*p* < 0.001), the ischemic stroke group (*Mdn* = 18) and the AD group (*Mdn* = 9) (*p* < 0.001), and the MCI group (*Mdn* = 18) and the AD group (*Mdn* = 9) (*p* < 0.001).

#### Orientation

*Post-hoc* analysis revealed statistically significant differences in orientation performance scores between the control group (*Mdn* = 12) and the AD group (*Mdn* = 8) (*p* < 0.001), the ischemic stroke group (*Mdn* = 12) and the AD group (*Mdn* = 8) (*p* < 0.001), and the MCI group (*Mdn* = 12) and the AD group (*Mdn* = 8) (*p* < 0.001).

#### Memory

*Post-hoc* analysis revealed statistically significant differences in memory performance scores between the control group (*Mdn* = 17) and the ischemic stroke group (*Mdn* = 10) (*p* < 0.001), the control group (*Mdn* = 17) and the AD group (*Mdn* = 3) (*p* < 0.001), and the MCI group (*Mdn* = 10) and the AD group (*Mdn* = 3) (*p* < 0.001). Controls also scored significantly higher than participants with MCI (*p* < 0.001).

#### Auditory comprehension

*Post-hoc* analysis revealed statistically significant differences in auditory comprehension performance scores between the control group (*Mdn* = 13) and the AD group (*Mdn* = 7) (*p* < 0.001), the ischemic stroke group (*Mdn* = 11) and the AD group (*Mdn* = 7) (*p* < 0.001), and the MCI group (*Mdn* = 11) and the AD group (*Mdn* = 7) (*p* < 0.001).

#### Reading comprehension

*Post-hoc* analysis revealed statistically significant differences in reading comprehension performance scores between the control group (*Mdn* = 12) and the AD group (*Mdn* = 7) (*p* < 0.001), the ischemic stroke group (*Mdn* = 11) and the AD group (*Mdn* = 7) (*p* < 0.001), and the MCI group (*Mdn* = 11) and the AD group (*Mdn* = 7) (*p* < 0.001).

#### Attention

*Post-hoc* analysis revealed statistically significant differences in attention performance scores between the control group (*Mdn* = 14) and the AD group (*Mdn* = 6) (*p* < 0.001), the ischemic stroke group (*Mdn* = 13.5) and the AD group (*Mdn* = 6) (*p* < 0.001), and the MCI group (*Mdn* = 13) and the AD group (*Mdn* = 6) (*p* < 0.001).

#### Problem solving

*Post-hoc* analysis revealed statistically significant differences in problem-solving performance scores between the control group (*Mdn* = 21) and the AD group (*Mdn* = 8) (*p* < 0.001), the ischemic stroke group (*Mdn* = 19) and the AD group (*Mdn* = 8) (*p* < 0.001), and the MCI group (*Mdn* = 18) and the Alzheimer’s disease group (*Mdn* = 8) (*p* < 0.001).

Table 6 presents the Kruskal–Wallis H statistics, degrees of freedom, *p*-values, and ε^2^ effect size estimates for the SCCAN-B total score and each performance scale.

**TABLE 6:** Kruskal–Wallis H test results for between-group comparisons.

Outcome	H (chi-square)	Df	*p-*value	ε ^2^
Oral expression	41.55	3	<0.001	0.45
Orientation	40.80	3	<0.001	0.44
Memory	55.91	3	<0.001	0.62
Auditory comprehension	38.15	3	< 0.001	0.41
Reading comprehension	29.36	3	<0.001	0.31
Writing	30.33	3	0.052	0.32
Attention	35.31	3	<0.001	0.38
Problem solving	35.23	3	<0.001	0.38
SCCAN-B total score	48.86	3	<0.001	0.54

Results from the Kruskal-Wallis H test for between group comparisons are presented for each SCCAN-B performance scale and the SCCAN-B total score.

### Preliminary screening classification

**FIGURE 2:**
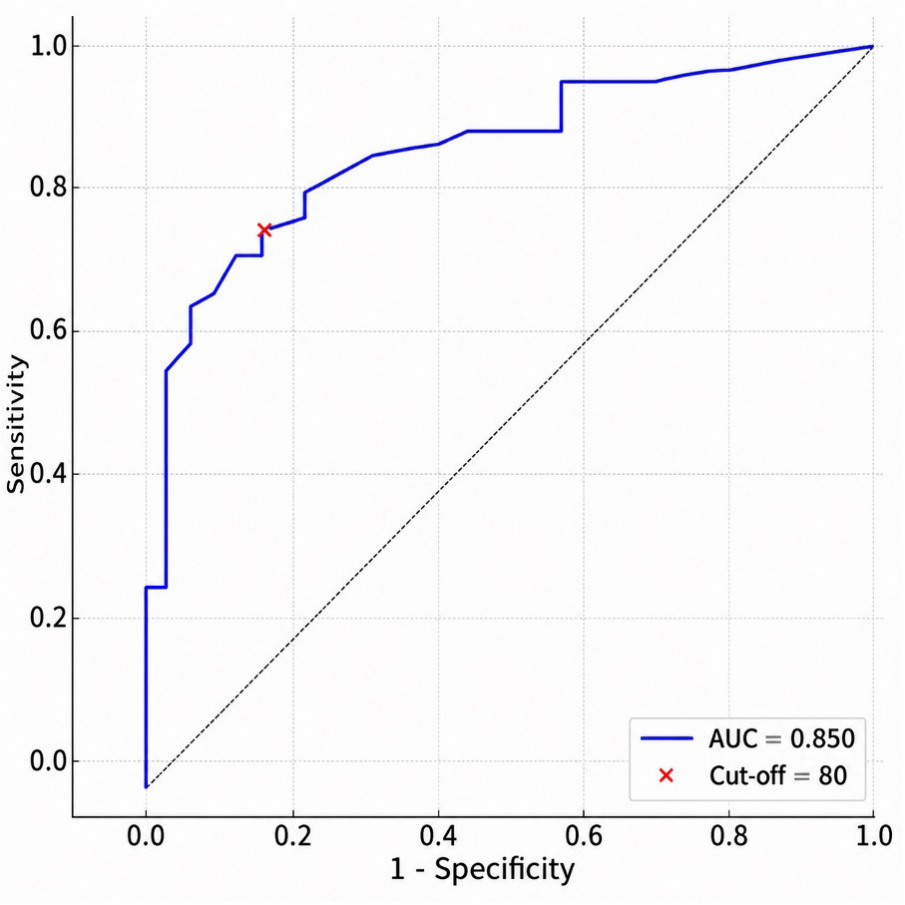
Receiver operating characteristic (ROC) curve for SCCAN-B total score. The area under the curve (AUC) was 0.850, indicating very good screening accuracy. A cut-off of 80 points is shown.

Preliminary screening classification was examined using ROC analysis. The control group was used as the reference group, and the main clinical groups (AD, MCI, and ischemic stroke) were classified as the comparison group. The area under the curve (AUC) was.850, indicating good preliminary classification performance. A SCCAN-B total score cut-off of 80 provided 70.7% sensitivity and 87.1% specificity in the present sample. The Youden index supported this threshold as the optimal sample-derived cut-off. Figure 2 shows the ROC curve. This threshold should be interpreted as preliminary and requires confirmation in larger independent samples.

### Cut-off classification by group

To further examine the preliminary clinical utility of the SCCAN-B threshold, participants were classified according to whether their SCCAN-B total score fell below or at/above the preliminary cut-off of 80. The distribution of participants below and at/above this threshold is presented by group in Table 7. This analysis was descriptive and should be interpreted as preliminary, because the cut-off was derived from the present sample and requires confirmation in larger independent cohorts.

**TABLE 7:** Classification of participants according to the preliminary SCCAN-B total score cut-off of 80.

Group	SCCAN-B < 80	Percent < 80	SCCAN-B ≥ 80	Percent ≥ 80
Control	4	12.9%	27	87.1%
MCI	8	61.5%	5	38.5%
AD	21	100.0%	0	0.0%
Ischemic stroke	12	50.0%	12	50.0%

MCI, Mild cognitive impairment; AD, Alzheimer’s disease; SCCAN-B, Bulgarian version of the Scales of Cognitive and Communicative Ability for Neurorehabilitation; and MMSE-B, Mini-Mental Status Examination.

As shown in Table 7, all participants with AD scored below the threshold, compared with 61.5% of participants with MCI and 50.0% of participants with ischemic stroke. In contrast, 87.1% of controls scored at or above the threshold. These findings should be interpreted cautiously because the cut-off was derived from the present sample and requires confirmation in larger independent cohorts.

## Discussion

This study provides preliminary initial-validation evidence for the Bulgarian version of the SCCAN. The SCCAN-B showed high internal consistency, strong association with MMSE-B scores, and significant known-groups differences between neurotypical controls and clinical neurological groups. These findings suggest that the SCCAN-B may be a clinically promising screening and profiling instrument for Bulgarian-speaking adults with neurological disorders.

The association between SCCAN-B and MMSE-B should be interpreted cautiously. The MMSE-B is a global cognitive screening instrument and does not directly assess cognitive-communicative abilities. Therefore, its association with SCCAN-B supports preliminary convergent validity with general cognitive status, but it does not establish comprehensive cognitive-communicative validity.

The observed association between SCCAN-B and MMSE-B may also have broader functional relevance. Global cognitive screening scores have been linked to instrumental activities of daily living, including financial capacity, which is clinically and legally important in older adults with neurocognitive disorders (Giannouli, 2023, 2024). However, SCCAN-B does not directly assess financial capacity, and the present study did not include direct instrumental activities of daily living (IADL) or legal-capacity measures. Therefore, the functional and medico-legal implications of SCCAN-B performance should be examined in future studies using dedicated IADL and financial-capacity instruments.

The current findings should not be interpreted as full normative standardization or definitive diagnostic validation. The ROC findings indicate preliminary screening classification performance in this sample, and the proposed cut-off requires confirmation in larger independent cohorts.

### Group and performance patterns

Analysis of median performance scale scores revealed distinct profiles across groups. Individuals in the control group showed consistently high scores across all domains. Participants in the MCI group performed at an intermediate level. Deficits were most marked in Memory, Attention, and Problem Solving, while Orientation, Writing, and Auditory Comprehension remained relatively preserved. Participants in the AD group had the lowest scores, with severe impairments in Memory, Orientation, Oral Expression, Attention, and Problem Solving. Deficits were evident across nearly all cognitive-communication domains. Ischemic stroke participants showed greater variability. Orientation and Oral Expression were relatively preserved, while Attention and Problem Solving were weaker. This pattern reflected lesion heterogeneity.

Taken together, these findings suggest that the SCCAN-B is sensitive to broad group-level differences in cognitive-communicative performance. However, the present data do not support definitive claims regarding disorder-specific profiles, and such interpretations require larger and diagnostically more homogeneous samples.

### Interpretation of cognitive-communicative profiles

*Post-hoc* Dunn tests with Bonferroni correction showed that control participants scored higher than clinical groups on nearly all domains. The most pronounced differences were observed between controls and individuals with AD. Participants with MCI demonstrated intermediate performance, with selective deficits primarily affecting memory and attention. The ischemic stroke group showed more variable performance patterns, differing from both controls and AD on several performance scales. Overall, these findings suggest that the SCCAN-B can detect clinically meaningful group-level differences in cognitive-communicative performance; however, the current sample size and clinical heterogeneity limit disorder-specific interpretation. Across clinical groups, memory impairment emerged as the most consistently affected domain. Individuals with AD demonstrated more widespread cognitive-communication deficits, while relatively preserved performance on reading and writing is consistent with evidence that these abilities are often affected later in the disease course. In contrast, the stroke group did not show marked language impairments, which is expected given that individuals with severe aphasia were excluded from study participation.

Participants in the AD group exhibited the most severe impairments, particularly in memory, orientation, oral expression, attention, and problem solving. This pattern aligns with previous findings indicating that episodic memory and semantic processing are among the earliest and most affected functions in AD (Dubois et al., 2014; Bayles et al., 2018). Prior SCCAN validation studies similarly demonstrated high sensitivity of memory and oral expression scales in dementia populations (Milman, 2003; Milman et al., 2008).

Participants with MCI performed between the control and AD groups, consistent with conceptual models describing MCI as a transitional stage between normal cognition and dementia (Albert et al., 2011; Petersen et al., 2014). Language abilities were relatively preserved, whereas selective impairments were evident in memory and attention. This pattern is consistent with earlier SCCAN research showing that these domains are particularly sensitive to pre-dementia cognitive changes (Milman and Holland, 2012).

Performance profiles in the ischemic stroke group were heterogeneous. Deficits were most frequently observed in attention and problem solving, whereas orientation and oral expression remained relatively intact. This variability likely reflects differences in lesion location and extent and is consistent with previous research on post-stroke cognitive–communicative outcomes (Hillis and Heidler, 2002; Lazar and Antoniello, 2008; Jaya et al., 2017). Although severe aphasia was an exclusion criterion, some participants demonstrated language impairments consistent with mild aphasia, underscoring the importance of multifactorial assessment in this clinical group.

Taken together, the present findings are broadly consistent with cognitive-communicative profiles reported in neurological populations and provide preliminary support for the clinical relevance of the SCCAN-B in Bulgarian practice. The results suggest sensitivity to shared and group-level patterns of cognitive-communicative impairment, particularly in AD and MCI. However, claims regarding disorder-specific profiles, diagnostic sensitivity comparable to the original English version, and general clinical applicability require confirmation in larger, independent, and diagnostically homogeneous samples.

### Clinical implications

The preliminary findings of this study have clinical relevance for Bulgarian neurological and neurorehabilitation practice. The SCCAN-B may help clinicians identify cognitive-communicative difficulties and describe broad profiles of relative strengths and weaknesses across domains such as memory, attention, orientation, oral expression, comprehension, reading, writing, and problem solving. This type of multi-domain profile may support referral decisions, rehabilitation planning, and interdisciplinary communication among neurologists, speech-language pathologists, neuropsychologists, and rehabilitation professionals.

The preliminary SCCAN-B total score cut-off of 80 may be clinically useful for identifying individuals who require more detailed cognitive-communicative or neuropsychological assessment. However, this threshold should not be interpreted as a definitive diagnostic boundary. It was derived from the present sample and requires confirmation in larger independent Bulgarian cohorts with age- and education-stratified normative data.

The present study also supports the feasibility of adapting a comprehensive cognitive-communication assessment for Bulgarian-speaking adults. However, the findings should not be taken as evidence of full cross-cultural standardization. Further work is required to confirm item-level equivalence, establish normative data, evaluate test-retest and interrater reliability, and compare SCCAN-B performance with broader neuropsychological and functional outcome measures.

Because the present study was cross-sectional, it does not provide direct evidence that the SCCAN-B can monitor longitudinal change. Future studies should examine whether SCCAN-B scores are sensitive to disease progression, rehabilitation effects, or changes in functional communication over time.

### Limitations and future directions

The current study is the first to report the use of the SCCAN-B in clinical practice, while also reporting preliminary psychometric properties. Thus, the present findings should be interpreted as preliminary initial-validation evidence rather than full normative standardization of the SCCAN-B. Several limitations should be considered. First, the sample size was modest, particularly within the MCI subgroup, which limits statistical power, subgroup interpretation, and the stability of cut-off estimates. Participants were recruited from a tertiary university hospital setting and therefore represent a convenience clinical sample rather than a population-representative Bulgarian sample. Larger multicenter studies with age- and education-stratified normative samples are required before definitive clinical cut-offs can be established.

Additionally, the clinical groups within the current study were heterogeneous. This is especially relevant for the ischemic stroke group, where lesion location, lesion laterality, aphasia subtype, handedness, and bilingualism were not systematically analyzed. Although participants with severe aphasia were excluded, no formal aphasia screening instrument was administered. Future studies should examine SCCAN-B performance in relation to lesion characteristics, aphasia diagnosis, and stroke subtype. In addition, the order of test administration was fixed rather than randomized. Although this approach ensured procedural consistency, possible order effects or fatigue effects cannot be fully excluded. Future studies should consider counterbalancing or randomizing the order of assessment instruments.

Although major clinical confounds were considered during neurological evaluation, standardized measures of depression, anxiety, fatigue, psychiatric symptoms, and functional status were not administered. These variables may influence cognitive and communicative performance and should be included in future validation studies.

The MMSE-B was the only external cognitive comparison measure administered systematically across participants. Although MMSE-B is a validated and widely used global cognitive screening instrument in Bulgaria, it does not directly assess cognitive-communicative abilities and should not be considered equivalent to a detailed neuropsychological or communication assessment. Future studies should compare SCCAN-B with broader neuropsychological batteries, communication-specific instruments, and functional outcome measures such as IADL measures.

An additional consideration relates to the translation and cultural adaptation of the SCCAN-B. Although the SCCAN-B was developed through a structured translation and cultural adaptation process including parallel forward translation, consensus review, back-translation, expert-panel evaluation, and pilot administration, further work is still needed to examine item-level equivalence in larger Bulgarian samples. Future studies should include more extensive cognitive interviewing, formal item-level analyses, and evaluation of differential item functioning across age, education, and diagnostic groups.

Finally, the study did not evaluate test-retest reliability, interrater reliability, sensitivity to longitudinal change, or treatment responsiveness. Future research should address these psychometric properties and examine whether SCCAN-B scores are sensitive to disease progression, rehabilitation effects, and changes in functional communication over time. Additional comparisons with established measures such as the Montreal Cognitive Assessment, the Boston Diagnostic Aphasia Examination, and other neuropsychological or communication-specific instruments would provide evidence of convergent and discriminant validity. This would also place the SCCAN-B in the broader context of international neuropsychological assessment tools.

## Conclusion

This study provides preliminary data for the initial-validation for the Bulgarian version of the SCCAN. The SCCAN-B showed high internal consistency, strong association with MMSE-B scores, and significant group-level differences between neurotypical controls and clinical neurological groups. ROC analysis indicated promising preliminary screening classification, with a SCCAN-B total score cut-off of 80 showing 70.7% sensitivity and 87.1% specificity in the present sample.

These findings support the SCCAN-B as a clinically promising screening and profiling instrument for Bulgarian-speaking adults with neurological disorders. However, the present study does not constitute full normative standardization and does not establish definitive diagnostic cut-offs. Larger multicenter studies with representative samples, age- and education-stratified norms, broader neuropsychological and functional comparison measures, and longitudinal designs are needed to confirm the psychometric properties and clinical utility of the SCCAN-B.

## Data Availability

The original contributions presented in the study are included in the article/Supplementary material, further inquiries can be directed to the corresponding author.
