# Supplementary material for: Preliminary validation of the Bulgarian SCCAN: initial reliability, validity, and screening accuracy across neurological populations
Source: Front Psychol. 2026 Jul 6;17:1846359. doi: 10.3389/fpsyg.2026.1846359 (PMC13381297; doi:10.3389/fpsyg.2026.1846359)
Supplement: Supplementary file 1 [file Table_1.docx]

**Supplementary Material 1
Summary of the Bulgarian Translation and Cultural Adaptation of the SCCAN-B**

The Bulgarian version of the Scales of Cognitive and Communicative Ability for Neurorehabilitation (SCCAN-B) was developed to support the clinical assessment of cognitive-communicative abilities in Bulgarian-speaking adults with neurological disorders.

The adaptation was guided by the biopsychosocial framework of the International Classification of Functioning, Disability and Health (ICF), which emphasizes body functions, activity, participation, and contextual factors. This framework is relevant to cognitive-communicative disorders because such disorders affect not only isolated language or cognitive functions but also communication effectiveness in everyday life.

The translation and cultural adaptation process was informed by international principles for cross-cultural test adaptation, including semantic, conceptual, cultural, and functional equivalence. The process included the following stages:

1. Parallel forward translation by two independent translators with experience in medical terminology and medical speech-language pathology.
2. Development of a consensus Bulgarian version through multidisciplinary expert discussion.
3. Back-translation to examine semantic adequacy and correspondence with the original version.
4. Expert-panel review for linguistic, conceptual, and cultural validity.
5. Pilot administration to examine comprehension, cultural applicability, and technical feasibility of the adapted tasks.

Culture-sensitive content was reviewed to identify material requiring modification for Bulgarian-speaking adults. The aim was to avoid literal translation where it would reduce clarity, cultural relevance, or functional equivalence. Adaptations were made to preserve the intended cognitive-communicative demands of the original tasks while ensuring that the material was familiar and accessible to Bulgarian patients.

Examples of culturally motivated adaptations included:

| ***Original content type*** | ***Bulgarian adaptation*** | ***Rationale*** |
| --- | --- | --- |
| ***Phonemic fluency letter cue*** | Adapted from the English cue to a Bulgarian letter cue | Preserves linguistic and phonological appropriateness in Bulgarian |
| ***Geographic orientation material*** | U.S.-specific geographic content adapted to Bulgarian geographic content | Preserves functional geographic relevance |
| ***Emergency number*** | U.S. emergency number adapted to 112 | Reflects the Bulgarian/EU emergency number |
| ***Medication-related content*** | U.S.-specific medication names adapted to names familiar in Bulgarian clinical practice | Preserves clinical and everyday familiarity |
| ***Idiomatic expression*** | Culturally specific English idiom replaced with a familiar Bulgarian idiomatic expression | Preserves pragmatic and figurative meaning |
| ***Date and currency formats*** | U.S. date format and dollars adapted to Bulgarian date format and Bulgarian leva | Preserves everyday functional relevance |

In written-response tasks, words were selected to be understandable, familiar, and appropriate for Bulgarian orthography and grammar. In orientation tasks, location-related questions were adapted so they could be used appropriately across Bulgarian clinical settings.

These adaptations did not change the structure of the SCCAN or the intended cognitive-communicative demands of the tasks. Their purpose was to preserve functional equivalence while improving linguistic clarity and cultural accessibility.

Because the SCCAN is a copyrighted instrument owned by PRO-ED, Inc., this supplementary material does not reproduce original or translated test items, scoring forms, stimulus materials, examiner instructions, or protected test content.
